# Effect of green-lipped mussel (*Perna canaliculus*) supplementation on faecal microbiota, body composition and iron status markers in overweight and obese postmenopausal women: a randomised, double-blind, placebo-controlled trial

**DOI:** 10.1017/jns.2023.41

**Published:** 2023-05-18

**Authors:** Maryam Abshirini, Jane Coad, Frances M. Wolber, Pamela von Hurst, Matthew R. Miller, Hong Sabrina Tian, Marlena C. Kruger

**Affiliations:** 1 Department of Human Nutrition, University of Otago, PO Box 56, Dunedin 9054, New Zealand; 2 School of Food and Advanced Technology, College of Sciences, Massey University, Palmerston North, New Zealand; 3 Centre for Metabolic Health Research, Massey University, Palmerston North, New Zealand; 4 School of Sport, Exercise and Nutrition, College of Health, Massey University, Auckland, New Zealand; 5 Cawthron Institute, Nelson, New Zealand; 6 Sanford Ltd., Auckland, New Zealand

**Keywords:** Body composition, Greenshell mussel, Gut microbe, Iron marker

## Abstract

The present study aimed to determine the effect of whole meat GSM powder on gut microbiota abundance, body composition and iron status markers in healthy overweight or obese postmenopausal women. This was a 3-months trial involving forty-nine healthy postmenopausal women with body mass index (BMI) between 25 and 35 kg/m^2^ who were randomly assigned to receive 3 g/d of either GSM powder (*n* 25) or placebo (*n* 24). The gut microbe abundance, serum iron status markers and body composition were measured at the baseline and the end of the study. The between-group comparison at the baseline showed a lower abundance of *Bacteroides* and *Clostridium* XIVa in the GSM group compared with the placebo (*P* = 0⋅04). At the baseline, the body fat (BF)% and gynoid fat% were higher in the GSM group compared with the placebo (*P* < 0⋅05). No significant changes were found in any of the outcome measures, except for ferritin levels that showed a significant reduction over time (time effect *P* = 0⋅01). Some trend was observed in bacteria including *Bacteroides* and *Bifidobacterium* which tended to increase in the GSM group while their abundance decreased or remained at their baseline level in the control group. Supplementation with GSM powder did not result in any significant changes in gut microbe abundance, body composition and iron markers compared with placebo. However, some commensal bacteria such as *Bacteroides* and *Bifidobacteria* tended to increase following supplementation with GSM powder. Overall, these findings can expand the knowledge surrounding the effects of whole GSM powder on these outcome measures in healthy postmenopausal women.

## Introduction

The link between obesity and chronic disorders onset and progression is well-documented. Low-grade chronic systemic inflammation, dyslipidaemia and altered glucose metabolism are recognised as major risk factors that provide pathophysiological mechanisms that underpin this association^(1)^. Furthermore, the role of the gut microbiota in several obesity-associated diseases has been demonstrated in humans^(2)^. Restoring the gut microbiota by dietary intervention has been demonstrated to attenuate the chronic inflammation in obese individuals^(3)^. Westernised diets characterised by a high intake of saturated fat and a low intake of fibre disrupt the microbial community and increase intestinal permeability^(4)^. Probiotics, prebiotics and nutritional supplements that are used for regulating the microbial communities are proposed as plausible therapeutic options for obesity-related disorders such as metabolic syndrome and osteoarthritis (OA)^(5,6)^.

*Perna canaliculus* is a New Zealand marine species known as Greenshell mussel™ (GSM) which has been extensively studied for its anti-inflammatory compounds such as omega-3 polyunsaturated fatty acids (*n*-3 PUFA)^(7)^. A novel potential prebiotic effect of GSM has been documented in an animal^(8)^ and a human clinical trial^(9)^. For example, the experimental study of rats fed with a high-fat/high-sugar (HFHS) diet demonstrated that adding whole GSM powder to the HFHS diet increased the caecum weight and caecal contents, which indicates the enhancement in colonisation of gut microbes. In addition, the production of short-chain fatty acids (SCFAs) such as propionic acid by gut microbes was significantly reduced in rats fed with the HFHS diet but increased when the rats were fed with GSM powder^(8)^. In a previous human study, supplementation with whole GSM extract powder or a glucosamine sulphate extract (3 g/d) for 3 months improved the gastrointestinal symptoms along with OA symptoms in patients with knee OA and showed a reduction in the abundance of the *Clostridium* and *Staphylococcus* species and an increase in *Lactobacillus*, *Streptococcus* and *Eubacterium* species in the gut microbe profile. Supplementation with GSM increased *Bifidobacterium* and *Enterococcus* in particular and decreased yeast species^(9)^. The similar effect of GSM and glucosamine on gut microbes suggests that glucosamine and similar compounds present in GSM powder provide a substrate for gut bacteria and account for its prebiotic activity, although utilisation of GSM by gut microbes has not been fully investigated. There are studies showing that supplementation with eicosapentaenoic acid (EPA) and docosahexaenoic acid (DHA) promoted the abundance of SCFAs producing bacteria such as *Bifidobacterium* and *Lactobacillus*^(10)^.

Obesity predisposes individuals to subclinical inflammation and concurrently reduces iron absorption and systemic iron availability from cellular iron stores^(11)^. It was shown that obese postmenopausal women had a moderate degree of iron deficiency compared with non-obese women as obese women had higher levels of soluble transferrin receptor (sTfR), even though no difference between ferritin levels was observed. The sTfR marker is an indicator of cellular iron status and is considered a useful clinical parameter for assessing iron status in these populations^(12)^. GSM contains high concentrations of both haem and non-haem iron along with iron absorption enhancers including cysteine-rich myofibrillar proteins, glycosaminoglycan (GAG) and omega-3 PUFA^(7,13)^. An *in vitro* study showed that GSM digestate enhanced non-haem iron uptake in a model of human intestinal epithelial (Caco-2) cells to a similar extent as beef^(14)^. Therefore, we hypothesised that GSM powder may have the potential to improve iron status in overweight/obese postmenopausal women.

The growth of fat and muscle mass is in synchrony during the younger stages of life; however, the balance can be impaired with aging causing the state of high-fat mass and relatively low muscle mass defined as sarcopenic obesity^(15)^. The effect of menopause on body fat accumulation and distribution is well-documented. It has been shown that postmenopausal women had 36 % greater trunk fat, 49 % more intra-abdominal fat and 22 % greater subcutaneous abdominal fat than premenopausal women^(16)^. There are several studies that have shown associations of sarcopenic obesity with increased risk of falls, fracture^(17)^, joint instability and malalignment of and knee OA, particularly in women. For example, a significantly increased risk of radiographic knee OA was observed among obese women and men and sarcopenic obese women, but not among sarcopenic obese men^(18,19)^. Thus, studying body composition in an intervention targeting obese women allows for the identification of a novel clinical marker, given the implication of sarcopenic obesity in the development of bone and joint disease. Recently, the novel weight-reducing potential of a GSM extract has been suggested from animal models^(20,21)^. Supplementation with a high-fat diet (HFD) enriched with GSM oil prevented body weight gain and was associated with decreased visceral fat mass in mice^(20)^. In a previous study of rats fed with the HFHS diet, the inclusion of GSM powder tended to increase the lean mass gain and decreased the fat mass gain in comparison to rats fed with only HFHS^(21)^.

This clinical intervention study aimed to determine whether GSM supplementation positively changes the faecal microbiota compared with placebo. A second objective of the study was to assess the effect of GSM supplementation on body composition and an iron status marker compared with the placebo in overweight or obese postmenopausal women.

## Method and materials

### Study participants

A total of forty-nine New Zealand women aged 55–75 years old who were at least 5 years post-menopause were included in the study. A further inclusion criterion was having a body mass index (BMI) of between 25 and 35 kg/m^2^. Exclusion criteria were formal diagnosis with OA or rheumatoid arthritis (RA), history of recent joint injury or trauma; having major chronic diseases such as liver or renal disease, diabetes mellitus or atherosclerosis; allergy to mussels or seafood; smoker or high intake of alcohol (>2 units per day); or use of any medication, antibiotics and supplements affecting the primary outcomes of the study within 3 months of beginning the trial. Other exclusion criteria were being on hormone replacement therapy or taking glucocorticoids drugs or non-steroidal anti-inflammatory drugs (NSAIDs) daily.

This study was conducted according to the guidelines laid down in the Declaration of Helsinki and all procedures involving human subjects/patients were approved by the Massey University Human Ethics Committee: Southern A, Application 20/03. Written informed consent was obtained from all subjects/patients. The study was registered with the Australian New Zealand Clinical Trials Registry with the number ACTRN12620000413921p.

### Study design

Participants who met the inclusion criteria were randomly assigned to either the GSM or placebo group, stratified by age (55–64, 65–75 years) and BMI (25–29⋅9 kg/m^2^ and obese: 30–35 kg/m^2^). The GSM group received six capsules of 500 mg each of whole GSM powder daily (equal to 3 g/d) for 12 weeks, and the control group received the same amounts of identical capsules of sunflower seed protein as a placebo. The flash-dried whole meat GSM powder was produced by Sanford Ltd (ENZAQ facility, Blenheim, New Zealand) using standard manufacturing processes. The GSM powder contained 41⋅4 % protein, 30⋅8 % carbohydrate, 10⋅1 % fat (EPA and DHA were 20⋅7 and 8 % total fatty acids, respectively), 10⋅7 % ash and 7 % moisture. The dose of 3 g/d was selected as it is achievable through diet (equivalent to 1–2 mussels per day). Sunflower seed protein used as a placebo (BP Bulk powders, Braeside, Melbourne, Australia) contained 24⋅3 % protein, 66⋅6 % carbohydrate, 3 % fat, 32⋅7 % ash and 4 % moisture. Sunflower seed protein was used as a placebo as a neutral source of protein and was selected to be relatively similar to GSM powder in respect to macronutrient composition and to be as inert and non-bioactive as possible. The amount of iron in GSM powder and placebo used in the study were 120 and 47 mg/kg for GSM powder and placebo, respectively. This equated to ~0⋅36 mg in 3 g GSM powder and ~0⋅14 mg in 3 g placebo.

Both GSM powder and placebo were encapsulated in hard-shell capsules by a commercial facility (Alaron, Nelson, NZ) and stored under nitrogen in the dark at room temperature or lower until use. The GSM and placebo capsules were identically matched in the shape, size and colour of the hard-shell encapsulant. Activated carbon sachets for absorbing moisture and odour were put in bottles to conceal any ‘fishy’ odour.

A randomisation list was generated by Excel and maintained by the project's supervising investigator, who did not interact with the study subjects or conduct the primary data analysis. Both the primary researcher and participants were blinded to treatment group allocation until all analyses were completed. Data were collected during participants’ visits at the baseline and the end of the study (week 12). All participants self-reported an omnivorous diet and the majority had the intake of probiotic products at least once a week in their diet before the study and were instructed to maintain their normal diet and physical activity throughout the trial. Recruitment, screening and data collection took place at the Human Nutrition Research Unit (HNRU) at Massey University, Palmerston North, New Zealand from August 2020 to September 2021.

### Demographic and anthropometric, and body composition measurements

The data on demographic characteristics were collected from participants at the baseline. The anthropometric measurements including body weight and standing height were measured using a beam balance to the nearest 0⋅2 kg and a stadiometer to the nearest 0⋅1 cm, respectively, at the baseline and the end of the study. BMI was calculated as weight (kg) divided by height squared (m^2^). Body composition measurements including whole and regional fat mass, lean mass and total body fat (BF)% were measured using a Hologic Horizon A, dual-energy X-ray absorptiometry (DXA) at the baseline and week 12. With data provided by the DXA scan, appendicular lean mass (aLM) was calculated as the sum of lean mass in arms and legs, assuming that lean mass in these areas is skeletal muscle. For the purpose of detecting sarcopenia, aLM index was calculated by adjusting for height and fat mass. Linear regression was applied to model the relationship between aLM and height and fat mass, and the residual of regression was used to identify the sarcopenic participants. Individuals were classified as sarcopenic if their value fell below the 20th percentile of the residual distribution. Since this method considers the fat mass, it has been recommended for identifying sarcopenia in women and overweight and obese individuals^(22)^.

### Assessment of physical activity

Physical activity was assessed by the New Zealand Physical Activity Questionnaire – Short Form (NZPAQ-SF)^(23)^. The NZPAQ has been validated by Boon *et al.*^(24)^, and physical activities were computed by the metabolic equivalent of task (METs)-min/week, which was calculated by the scoring protocol of the International Physical Activity Questionnaire (IPAQ) for continuous score^(25)^.

MET values and formula for calculation of MET-minutes were assessed and used as below:

- Walking MET-minutes/week at work = 3⋅3 × walking minutes × walking days at work.
- Moderate MET-minutes/week at work = 4⋅0 × moderate intensity activity minutes × moderate intensity days at work.
- Vigorous MET-minutes/week at work = 8⋅0 × vigorous intensity activity minutes × vigorous.
- Total work MET-minutes/week = sum of walking + moderate + vigorous MET-minutes/week scores at work.

### Dietary intake assessment

Each participant's daily nutrient intake from the diet was measured using a 3-d food record including two weekdays and one weekend day at the midpoint of the trial. The 3-d food record has been recommended and considered the ‘gold standard’ for dietary assessment. Instructions on how to accurately complete the food record were provided^(26)^. The brand name of food products, recipes and food preparation were recorded. Each participant's nutrient intake was calculated using Foodworks 9 Professional, Xyris Software.

### Faecal sample collection, DNA isolation and gut microbe quantification

Participants were provided with a stool sample collection kit at the baseline and the end of the study. Each kit contained a container, an anaerobic bag with anaerobic sachet plus a freezer pack. Samples were delivered to the HNRU and stored at −80 °C. Bacterial DNA was extracted using an Isolate Faecal DNA kit (Bioline, NSW, Australia) at the completion of the study. Briefly, 250 μl of each homogenised faecal sample was mixed with 750 μl of lysis buffer in a tube containing bashing beads and processed with a beater at a maximum speed for 10 min to disrupt the cells following the manufacturer's instructions. The faecal DNA was eluted from the column and stored at −80 °C for later analysis. The concentration and purity of extracted DNA were measured using a Nanodrop 2000 Spectrophotometer (Thermo Fisher Scientific, USA). The quantitative real-time polymerase chain reaction (qRT-PCR), using SYBR™ Green Master Mix, was performed on a LightCycler® 480 Real-Time PCR instrument (Roche Applied Science). The specific primers for bacteria are presented in Table 1. The q-PCR was carried out in a total volume of 20 μl containing 10 μl 2× LightCycler® 480 SYBR Green I Master (Roche Diagnostic, Indianapolis, IN, USA), 1 μl of each primer (forward and reverse) and 3–8 μl of target DNA (due to high variability in the concentration of extracted DNA); nuclease-free water was added to reach a final volume of 20 μl. For purposes of standardisation, depending on the DNA concentration of the sample, 3, 5 and 8 μl of DNA were used for samples with concentrations >90, 90–10 and <10 ng/μl, respectively.

**Table 1.** Primers used for quantitative real-time polymerase chain reaction (qRT-PCR)

Bacteria	Forward primer	Reverse primer
*Bifidobacterium*	TCGCGTCYGGTGTGAAAG	CCACATCCAGCRTCCAC
*Lactobacillus*	AGCAGTAGGGAATCTTCCA	CACCGCTACACATGGAG
*Akkermansia muciniphila*	CAGCACGTGAAGGTGGGGAC	CCTTGCGGTTGGCTTCAGAT
*Bacteroides*	GAAGGTCCCCCACATTG	CAATCGGAGTTCTTCGTG
*Clostridium* cluster IV	ACAATAAGTAATCCACCTGG	CTTCCTCCGTTTTGTCAA
*Clostridium* cluster XIVa	AAATGACGGTACCTGACTAA	CTTTGAGTTTCATTCTTGCGAA

### Biochemical analysis

Non-fasted blood samples were collected at the baseline and the end of the study to measure iron status markers including serum iron, transferrin, ferritin, sTfR, iron saturation, iron binding as well as C-reactive protein (CRP) levels as a marker of inflammation. All biomarkers were analysed at Medlab Central-Palmerston North, New Zealand. In this study, participants were defined as iron-deficient or depleted if they had ferritin <30 μg/l or sTfR was >1⋅76 mg/l. Systemic inflammation was defined as a CRP concentration >5 mg/l. These values are according to hospital-based clinical laboratory reference, and previously published cut-offs^(27,28)^.

### Compliance

Compliance was checked at week 6 and the end of the study by examining the participant's records of their daily intake of study supplements. Compliance was assessed using cumulative capsule counts at the completion of the study, and adherence was measured as a percentage: (number of capsules provided − number of unused capsules)/number of capsules provided ×100. Adherence below 80 % was considered a protocol violation.

### Statistical methods

The microbial analyses were secondary objectives of the original trial, and the sample size calculation was not based on detecting significant changes in either gut microbiota abundance, body composition parameters or iron status markers. In brief, the sample size was based on urinary C-telopeptide of type II collagen (CTX-II)/creatinine and serum cartilage oligomeric matrix protein (COMP) as the primary outcomes of the study. The sample size was calculated to detect a 20 % difference between the groups using the standard deviation from the latest lab result. For urinary CTX-II/creatinine a sample size of 60 and for serum COMP a sample size of 17 were required to detect a 20 % difference between groups with a power of 95 %. The serum COMP required a small sample size, thus the average of two outcomes was used for the sample size of 48 (*n* 24 per group). Finally, a total sample size of 55 was needed to allow for at ~10 % potential dropout rate (*n* 27–28 per group). A previous study among knee OA patients investigating the effect of whole GSM powder on the gut microbiome using similar dose and duration, demonstrated changes in gut microbiota profile in relatively smaller samples sizes (*n* 38)^(9)^. Data analyses were performed with SPSS software IBM SPSS version 26.0 (Armonk, NY). Analyses were done on the dataset from those who completed at both timepoints (baseline and endpoint). Variables were checked for normality using the Kolmogorov–Smirnov and Shapiro–Wilk tests and data that were not normally distributed were log-transformed. The data were reported as mean ± standard deviation (sd) for normally distributed data, and as median (25th and 75th percentiles) for non-normally distributed data, and as frequencies for categorical data. The between-group differences were tested by using the Student's *t* test and the Mann–Whitney *U* test for parametric and non-parametric data, respectively. For categorical variables, the differences at the baseline were assessed by *χ*^2^, and by Fisher's exact test where >20 % of cells had an expected count below 5. Two-way repeated measures analysis of variance (ANOVA) was used to examine differences within each group over time (pre- *v.* post-intervention) and between the groups (GSM *v.* placebo). The interactions between treatments and time indicate differences in efficacy. The level of significance was *P* < 0⋅05.

## Results

### Baseline characteristics

Out of fifty-five participants who enrolled in the study, six participants withdrew during the trial due to health issues or being non-compliant. A total of forty-nine participants (GSM, *n* 25 and placebo, *n* 24) completed the study. The general characteristics and percentage of participants with iron depletion and sarcopenia are shown in Table 2. The prevalence of sarcopenia was 13 % (*n* 3) in placebo and 24 % (*n* 6) in the GSM group. At the baseline, only one participant from the GSM group had a serum ferritin <30 μg/l, and three participants (placebo, *n* 2 and GSM, *n* 1) were detected with elevated sTfR >1⋅76 mg/l. Systemic inflammation assessed by CRP >5 mg/l was present in six women (12⋅2 %) at the baseline (GSM, *n* 3 and placebo, *n* 3). There were no differences between treatment groups for any of the baseline measurements.

**Table 2.** Baseline characteristics of participants who completed the study across the treatment groups (*n* 49)

Characteristic	Placebo (*n* 24)	GSM (*n* 25)	*P*-value
Demographic/anthropometric measurements			
Age (year)	62⋅9 ± 5⋅4	64⋅2 ± 5⋅1	0⋅3
Height (cm)	164⋅7 ± 6⋅4	164⋅8 ± 7⋅1	0⋅8
Weight (kg)	73⋅8 (68⋅2, 88⋅6)	77⋅2 (68⋅5, 86⋅2)	0⋅9
BMI (kg/m^2^)	29⋅1 ± 4⋅2	29⋅1 ± 3⋅3	0⋅9
Having sarcopenia^a^, *n* (%)	3 (13)	6 (24)	0⋅3
Physical activity (MET-minutes/week)	751 (318, 2373⋅7)	764 (287, 1483)	0⋅1
Iron status markers			
Serum ferritin^b^ <30 μg/l, *n* (%)	0 (0)	1 (4)	0⋅5
Serum sTfR^a^ >1⋅76 mg/l, *n* (%)	2 (8⋅3)	1 (4)	0⋅6
Serum CRP^a^ >5 mg/l, *n* (%)	3 (12⋅5)	3 (12)	0⋅9

BMI, body mass index; MET, metabolic equivalent of task; sTfR, soluble transferrin receptors; CRP, C-reactive protein.

Values are presented as mean ± standard deviation and median presented as (25th and 75th percentile) for normally distributed and non-normally distributed variables, respectively, and *n* (%) for categorical variables for which the percentage within each treatment group is reported.

a Data were available for forty-eight subjects (placebo, *n* 23 and GSM, *n* 25).

b Cut-off value according to hospital-based clinical laboratory reference range and previously published.

The daily nutrient intake of participants is presented in Table 3. The daily intake of iron from the diet was 11⋅2 ± 5 mg in the GSM and 16⋅2 ± 17⋅8 mg in the placebo group, with no differences between the groups. Both groups were comparable in the term of energy and macronutrient intakes.

**Table 3.** The daily energy and nutrients intake from the diet (without supplements) of participants across treatment groups

Daily energy and nutrients intake	Placebo (*n* 24)	GSM (*n* 21)
Energy (kJ)	9339⋅1 ± 3322⋅1	9030 ± 4469⋅3
Protein (g)	99⋅9 ± 52⋅3	93⋅04 ± 31⋅2
Carbohydrate (g)	203⋅5 ± 91⋅5	193⋅6 ± 94⋅6
Fat (g)	101⋅1 ± 44⋅5	100⋅9 ± 68⋅8
SFA (g)	38⋅9 ± 18	40⋅2 ± 30⋅2
MUFA (g)	35⋅6 ± 22	35⋅4 ± 22⋅8
PUFA (g)	14⋅7 ± 7⋅2	14⋅8 ± 15⋅2
EPA (g)	0⋅04 ± 0⋅05	0⋅03 ± 0⋅04
DHA (g)	0⋅04 ± 0⋅06	0⋅03 + 0⋅08
Calcium (mg)	1442⋅3 ± 1797⋅1	1061⋅0 ± 509⋅2
Sodium (mg)	2413⋅7 ± 758	2282⋅0 ± 910⋅4
Potassium (mg)	7524⋅0 ± 14 524	5357⋅4 ± 1178⋅8
Iron (mg)	16⋅2 ± 17⋅8	11⋅2 ± 5⋅0
Zinc (mg)	13⋅8 ± 9⋅5	11⋅5 ± 5⋅9
Magnesium (mg)	814⋅0 ± 1636⋅1	732⋅2 ± 171⋅9
Dietary fibre (g)	28⋅6 ± 13⋅0	24⋅7 ± 8⋅2

SFA, saturated fatty acids; MUFA, monounsaturated fatty acids; PUFA, polyunsaturated fatty acids; EPA. eicosapentaenoic acid; DHA, docosahexaenoic acid.

### Evaluation of treatment on gut microbiota abundance

**Fig. 1.**
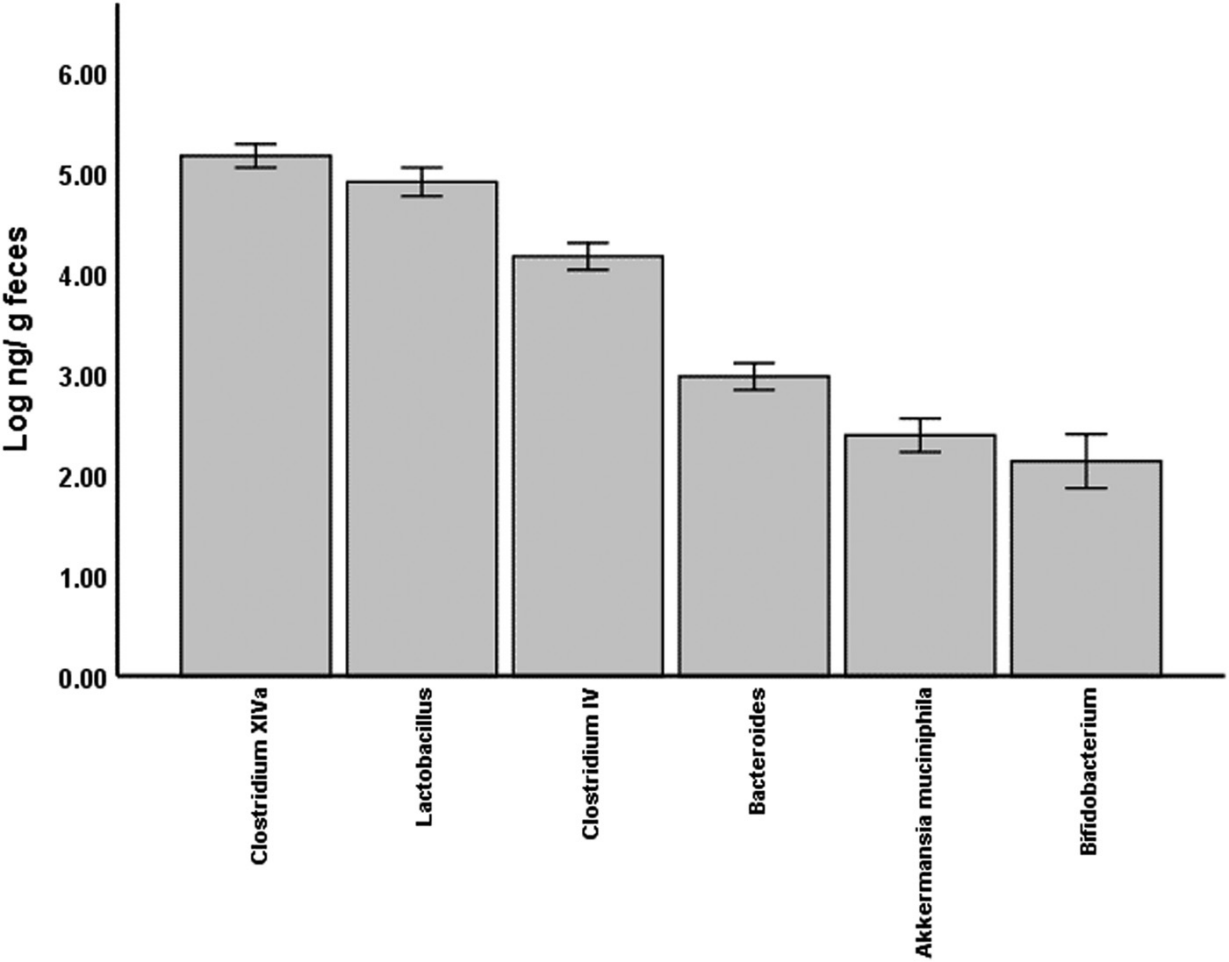
The abundance (mean ± se) of *Clostridium* XIVa, *Lactobacillus*, *Clostridium* IV, *Bacteroides*, *Akkermansia muciniphila* and *Bifidobactrian* from the baseline faecal samples of the overall study population (*n* 49).

**Fig. 2.**
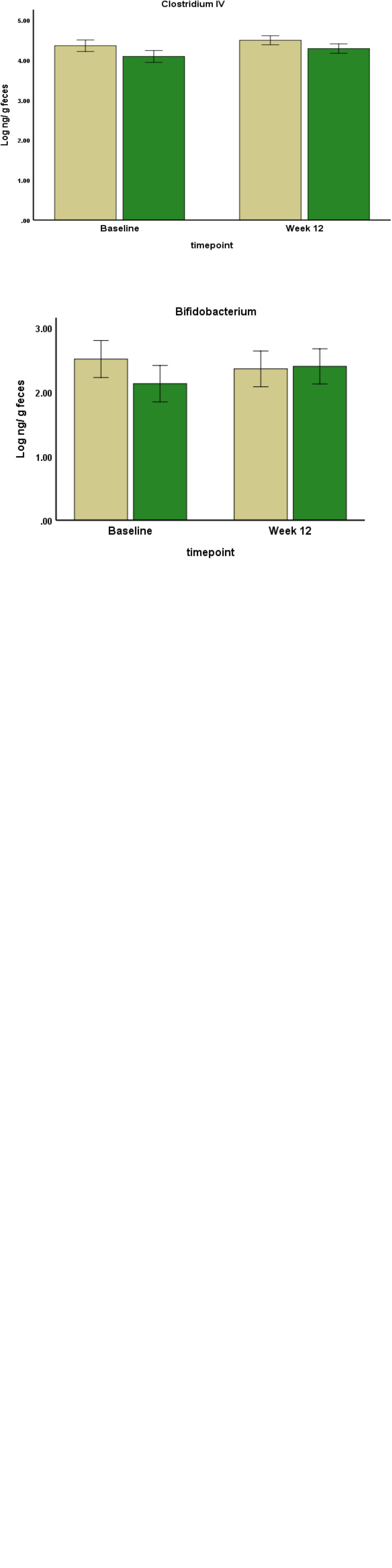
The abundance of bacteria at the baseline and the end of the study were measured by real-time PCR. Data are presented in log scale ng/g faeces (mean ± se). The two-way repeated measure ANOVA did not show any significant change within the groups (time effect) or any significant change from the baseline between the groups (time and treatment interaction effect). Statistical differences between the groups at each timepoint were assessed using the Student's *t* test. *Indicates the significance at *P* < 0⋅05.

The baseline abundance of each selected bacteria is demonstrated in Fig. 1. The *Clostridium* XIVa and *Lactobacillus* had the highest abundance (mean ± se; 5⋅1 ± 0⋅1 and 4⋅9 ± 0⋅1 log ng/g faeces, respectively), followed by *Clostridium* IV and *Bacteroides*, while *Akkermansia muciniphila* and *Bifidobacterium* had the lowest abundance among all bacteria (2⋅5 ± 0⋅1 and 2⋅3 ± 0⋅3 log ng/g faeces). As demonstrated in Fig. 2, the between-group comparison at the baseline showed a significant difference in the abundance of some of the bacteria which were lower in the GSM group than the placebo group. These bacteria included *Bacteroides* (2⋅8 ± 0⋅75 *v.* 3⋅2 ± 0⋅47 log ng/g faeces, *P* = 0⋅04), *Clostridium* XIVa (4⋅9 ± 0⋅58 *v.* 5⋅3 ± 0⋅57 log ng/g faeces, *P* = 0⋅04) and *A. muciniphila* (2⋅2 + 0⋅99 *v.* 2⋅7 + 0⋅6 log ng/g faeces, *P* = 0⋅08). After 12 weeks of supplementation with GSM, there was a relative increase in the abundance of *Bacteroides*. The significant difference between the groups disappeared at the end of the intervention, while the abundance of this bacteria was maintained in the placebo group. *Bifidobacterium* and *A. muciniphila* abundance tended to increase slightly after GSM supplementation, while it was maintained or reduced in the placebo group. The difference in *Clostridium* XIVa abundance between the groups was significant at the baseline and it disappeared at the end of the study. However, *Clostridium* XIVa and other bacteria including *Lactobacillus*, *Clostridium* IV did not alter and remained at their baseline level during the intervention periods. Overall, no statistically significant changes in the abundance of bacteria were noted during the intervention (time and treatment interaction effect) assessed by two-way repeated measure ANOVA.

### Evaluation of treatment on iron markers and CRP level

The baseline, endpoint and % change from baseline in iron markers of study population across the treatment groups are presented in Table 4. Iron status markers and CRP level did not show any significant differences between the groups at the baseline, the end of the study and in % change from baseline using the Student's *t* test. However, a significant time effect was noted for ferritin levels which showed a significant reduction in the GSM group over time (144⋅8 ± 91⋅5 *v.* 125⋅1 ± 71⋅5, *P* = 0⋅01).

**Table 4.** The baseline, endpoint and % change from the baseline of iron markers and CRP level over the 12 weeks of study across treatment groups (*n* 47)

	Reference range	Placebo (*n* 23)	GSM (*n* 24)	*P*-value*
Serum iron (μmol/l)	10–30			
Baseline		15⋅9 ± 3⋅8	15⋅4 ± 3⋅9	0⋅6
Endpoint		15⋅5 ± 50⋅2	14⋅6 ± 3⋅5	0⋅9
% Change		−0⋅9 ± 35⋅5	−0⋅1 ± 31⋅3	0⋅9
Transferrin (g/l)	2–3⋅2			
Baseline		2⋅6 ± 0⋅3	2⋅4 ± 0⋅2	0⋅06
Endpoint		2⋅5 ± 0⋅3	2⋅5 ± 0⋅2	0⋅3
% Change		−0⋅09 ± 8⋅3	2⋅7 ± 6⋅0	0⋅1
Ferritin (μg/l)	30–350			
Baseline		118⋅9 ± 66⋅6	144⋅8 ± 91⋅5	0⋅2
Endpoint		111⋅0 ± 63⋅3	125⋅1 ± 71⋅5 **	0⋅4
% Change		−4⋅9 ± 20⋅6	−6⋅5 ± 30⋅5	0⋅5
Iron saturation (%)	20–50			
Baseline		27⋅0 ± 6⋅5	27⋅7 ± 6⋅9	0⋅7
Endpoint		26⋅4 ± 8⋅8	25⋅7 ± 6⋅9	0⋅6
% Change		1⋅1 ± 34⋅1	−2⋅3 ± 8⋅6	0⋅7
sTfR (mg/l)	0⋅83–1⋅76			
Baseline		1⋅3 ± 0⋅3	1⋅1 ± 0⋅2	0⋅09
Endpoint		1⋅3 ± 0⋅3	1⋅2 ± 0⋅3	0⋅1
% Change		0⋅2 ± 8⋅6	1⋅9 ± 16⋅0	0⋅6
Iron binding (μmol/l)	50–80			
Baseline		59⋅4 ± 7⋅01	56⋅0 ± 6⋅4	0⋅08
Endpoint		59⋅3 ± 6⋅79	56⋅1 ± 9⋅1	0⋅1
% Change		0⋅1 ± 8⋅2	0⋅6 ± 13⋅6	0⋅8
CRP (mg/l)	<5			
Baseline		2⋅1 ± 2⋅3	2⋅8 ± 2⋅1	0⋅2
Endpoint		2⋅5 ± 3⋅1	3⋅3 ± 2⋅7	0⋅3
% Change		35⋅6 ± 10	53⋅5 ± 20	0⋅7

* The difference between the groups was compared using the Student's *t* test.

** A significant time effect was noted for ferritin using two-way repeated measure ANOVA (*P* = 0⋅1).

The baseline mean ± sd concentration of CRP was in the normal range (2⋅4 ± 2⋅2) mg/l. The level of CRP increased during the study in both groups. The CRP >10 mg/l is defined as increased inflammation and used in iron studies as level of inflammation affecting ferritin level. Because none of the participants had CRP above this level, therefore correction of ferritin for inflammation was not necessary. Overall, there was no evidence of significant change in the iron status marker and CRP levels during the intervention using two-way repeated measure ANOVA.

### Evaluation of treatment on body composition parameters

The baseline, endpoint and % change from baseline in body composition parameters across the treatment groups are shown in Table 5. Body weight, lean mass, fat mass, trunk fat and android fat% were not different between the groups at the baseline and the end of the study, while BF% and gynoid fat% were significantly different between the two groups at the baseline and were significantly higher in the GSM group compared with placebo (BF%: 44⋅9 ± 4⋅1 *v.* 42⋅1 ± 5⋅1, *P* = 0⋅04, gynoid fat%: 46⋅7 ± 4⋅2 *v.* 43⋅2 ± 4⋅9, *P* = 0⋅01, respectively). However, the difference for fat% at the end of the study was no longer significant (45⋅2 ± 3⋅9 *v.* 42⋅9 ± 5⋅3, *P* = 0⋅09) with a greater increase in BF% in the placebo than the GSM (% change from baseline: 1⋅0 ± 2⋅6 *v.* 0⋅60 ± 3⋅0, *P* = 0⋅6). With regard to gynoid fat%, the difference between the two groups at the end of the study remained significant (46⋅9 ± 4⋅3 *v.* 43⋅7 ± 5⋅0, *P* = 0⋅03). Moreover, trunk fat had a higher increase during the study in the placebo *v*. the GSM group (% change from baseline: 1⋅1 ± 4⋅2 *v.* 0⋅4 ± 5⋅3, *P* = 0⋅4). Overall, no significant difference in % change from baseline between treatment groups for any of the parameters was observed.

**Table 5.** The baseline, endpoint and % change from the baseline in body composition parameters over the 12 weeks of study across treatment groups (*n* 48)

	Placebo (*n* 23)	GSM (*n* 25)	*P*-value*
Body weight (kg)			
Baseline	78⋅9 ± 12⋅8	79⋅4 ± 11⋅7	0⋅8
Endpoint	79⋅0 ± 12⋅7	79⋅4 ± 12⋅7	0⋅9
% Change	0⋅1 ± 1⋅9	0⋅12 ± 1⋅0	0⋅9
Lean mass (kg)			
Baseline	43⋅7 ± 4⋅9	42⋅5 ± 6⋅3	0⋅4
Endpoint	43⋅3 ± 5⋅0	42⋅3 ± 6⋅1	0⋅5
% Change	−0⋅4 ± 2⋅3	−0⋅1 ± 0⋅9	0⋅5
Fat mass (kg)			
Baseline	34⋅1 ± 9⋅1	36⋅5 ± 6⋅8	0⋅2
Endpoint	35⋅0 ± 9⋅0	36⋅8 ± 6⋅7	0⋅4
% Change	1⋅2 ± 3⋅4	0⋅8 ± 4⋅2	0⋅7
Body fat%			
Baseline	42⋅1 ± 5⋅1	44⋅9 ± 4⋅1	0⋅04
Endpoint	42⋅9 ± 5⋅3	45⋅2 ± 3⋅9	0⋅09
% Change	1⋅0 ± 2⋅6	0⋅6 ± 3⋅0	0⋅6
Trunk fat (kg)			
Baseline	16⋅1 ± 2⋅4	17⋅2 ± 4	0⋅3
Endpoint	16⋅3 ± 4⋅2	17⋅3 ± 3⋅8	0⋅5
% Change	1⋅1 ± 4⋅2	0⋅4 ± 5⋅3	0⋅4
Android fat%			
Baseline	40⋅8 ± 5⋅7	42⋅1 ± 5⋅3	0⋅4
Endpoint	41⋅2 ± 5⋅6	42⋅5 ± 5⋅0	0⋅5
% Change	1⋅0 ± 3⋅8	1⋅2 ± 4⋅1	0⋅8
Gynoid fat%			
Baseline	43⋅2 ± 4⋅9	46⋅7 ± 4⋅2	0⋅01
Endpoint	43⋅7 ± 5⋅0	46⋅9 ± 4⋅3	0⋅03
% Change	0⋅99 ± 2⋅9	0⋅52 ± 3⋅2	0⋅6

* The difference between the groups was compared using the Student's *t* test. The % change from the baseline was measured using analysis of covariance (ANCOVA) adjusted for the baseline level of fat% as a covariate.

### Safety and adverse events

There was no evidence of a change between groups for their lipid profile, liver enzymes and kidney function tests. The majority of participants were tolerant without any side effects. In contrast, five subjects in the GSM group and two subjects in the placebo group reported adverse events including moderate indigestion, reflux, mild abdominal pain and nausea.

## Discussion

The results of our study showed that the abundance of some of bacteria, particularly *Bacteroides*, *A. muciniphila* and *Bifidobacterium*, tended to moderately increase after 12 weeks of supplementation with whole GSM powder compared with placebo. With respect to iron status markers, no significant changes were noted except for ferritin levels which significantly decreased over time in the GSM group. No significant changes were observed in body composition; however, participants in the GSM group showed a less increase in BF%, and regional trunk fat and gynoid fat%, although none of these changes reached significance.

The baseline levels of some gut microbes including *Bacteroides* and *Clostridium* XIVa were different between the GSM and placebo groups which could be due to differences in diet and intake of probiotics or prebiotics as most participants of the study participants reported intake of some type of probiotic products at least once a week in their diet. In addition, other factors such as use of antibiotics and age can modulate the balance of gut microbiota^(29)^.

So far, little is known about the effect of GSM on the gut microbiome. Presently, there is only one human and one rat study that directly evaluated the effect of GSM powder on gut microbiota. Our results revealed a slight increase in the abundance of *Bacteroides* following GSM supplementation. Other bacteria including *Bifidobacterium* and *A. muciniphila* also tended to increase in the group received GSM powder. *Bacteroides* is the most abundant bacteria in the human gut and uses glycan as its main source of energy^(30)^. Chondroitin sulphate and glucosamine sulphate have very low absorption rates in the small intestine (5–15 %), and reach the colon where more than 50 % are degraded by the gut bacteria and then absorbed^(31)^. This suggests that the gut microbiota play a crucial role in the bioavailability of chondroitin and glucosamine sulphate to the host^(32)^. Optimising gut microbiota may improve the therapeutic efficacy of GSM and help to ameliorate the OA condition^(33)^. Previously, it was shown that supplementation with glycosaminoglycans (GAG) such as chondroitin sulphate increased the abundance of *Bacteroides*^(34)^. Similar to this result, a previous human study found increases in the abundance of *Bacteroides* and *Bifidobacterium* following supplementation with GSM powder (3 g/d) for 12 weeks^(9)^. Thus, it is possible that GSM powder which contains GAG provides substrates for these bacteria and supports their growth, which subsequently results in better absorption and enhanced protection of the gut barrier.

It should be noted that *Bifidobacterium* had the lowest abundance compared with other bacteria in our study. This could be because our study population was aged, and the lowest abundance of *Bifidobacterium* (~5–10 % relative abundance) has been confirmed among the elderly population. Other factors such as obesity, diabetes and allergies have been associated with a lower number of this bacteria at various stages of life^(35,36)^.

*A. muciniphila* is another beneficial bacterial species which tended to increase after GSM supplementation in this study. These bacteria produce mucin-degrading enzymes and use mucin in the mucus layer of the intestine as a source of nitrogen and carbon. It is known for its beneficial effect on obesity and improving insulin sensitivity and blood cholesterol^(37,38)^. Supplementation with omega-3 PUFA, which is the main type of fatty acid present in GSM^(39)^, has been shown to promote the abundance of this bacteria and to reduce gut inflammation^(40)^. However, in a previous study, the abundance of this bacteria decreased in rats fed with an HFHS diet and adding GSM to the diet did not change this pattern^(8)^. It should be taken into account that the relationship between this bacteria and the host is closely influenced by the energy intake from the diet and glucose and lipid metabolism^(41)^; in the rat study cited, the diet was abnormally high in both sugar and fat. In addition, the difference between the human and rat digestive tract and variation in bacteria could be part of the reason. The bacteria assessed in the rat study came from the caecum, which is a part of the gastrointestinal system that in rats containing bacteria capable of digesting the cellulose; however, the caecum in humans do not possess these bacteria.

*Clostridium* IV, also called *C. leptum*, and cluster XIVa are the main cluster of *Clostridium* in healthy individuals, representing 10–40 % of the total bacteria and contributing to the regulation of intestinal homeostasis^(42)^. In our study, the abundance of *Clostridium* XIVa and IV remained stable in both the GSM and placebo groups over the study period. However, a previous study reported a notable reduction in *Clostridium* after GSM supplementation^(9)^. It must be noted that in that study, the majority of participants in both arms (GSM and glucosamine) had moderate to severe gastrointestinal complaints with a high proportion of pathogenic species of *Clostridium* such as *C. innocuum* and *C. tertium*^(9)^. Furthermore, the beneficial effect of *Clostridium* has mainly been related to the treatment of intestinal autoimmune disease such as colitis and allergic diarrhoea via inducing the expansion and differentiation of regulatory T lymphocytes (Treg cells) and producing SCFAs such as butyrate^(43,44)^. However, participants of the current study were healthy and without gastrointestinal symptoms; therefore, it appears that the beneficial effect of GSM powder on *Clostridium* depends on the intestinal state of the host and differs across species.

In this study, the diversity of gut microbiota was not assessed as it was unlikely to change based on previous data that showed supplementation with GSM^(9)^ or chondroitin sulphate^(45)^ did not affect the biodiversity of gut microbiota, but it altered the abundance of individual genera of bacteria.

To our knowledge, this is the first study to investigate the effect of GSM powder on body composition in human subjects. Measurement of body composition is preferable to weight and BMI, due to the fact that body composition accounts for changes in fat, water and muscle mass as opposed to overall weight change^(46)^. In the present study, increases in the BF% over the study period appeared to be less in the GSM group than the placebo group, suggesting that GSM may have ameliorated body fat gain. This is consistent with the study on rats fed with an HFHS diet, in which adding whole GSM powder to the diet resulted in less body fat gain^(21)^. Similarly, reductions in body weight gain as well as inflammatory cytokines, serum triglycerides and mRNA expression of leptin were observed in rats fed with an HFD enriched with freeze-dried blue mussel powder^(47)^. Another study on male mice showed that an HFD containing 63 % of fat from GSM oil was able to prevent body weight gain and decreased the visceral fat mass^(20)^. The current evidence on the weight-reducing effect of GSM is limited to animal models and the lack of effect on body composition in the current study is not surprising and was anticipated, given that consuming 3 g GSM powder per day for 3 months may not be sufficient to result in changes in body weight or body composition. The recommended gold-standard for clinical practice in achieving weight loss and improving body composition includes several lifestyle-based interventions such as calorie restriction and regular physical activity for ≥6 months^(48)^. This approach has been found effective in a population of middle-aged, white women with obesity similar to our study population^(49)^. Furthermore, given that our participants were free living, and their dietary intake was assessed based on self-reported food intake and not controlled as in animal models, it could explain why the anti-obesity effects were not obvious in this study.

No significant changes were observed in iron status markers, except for a decrease in ferritin levels and an increase in transferrin levels in the GSM group, although no significant differences were observed between groups. This lack of change could be due to the fact the majority of participants had an adequate iron status and none of them were experiencing iron deficiency or anaemia with only 8 % having a small or depleted body iron reserve at the baseline. Moreover, the mean dietary iron intake of participants was above the 8 mg/d which is the recommended dietary allowance in women over 50 years^(50)^. Further studies targeting participants with inadequate dietary iron intake or iron depletion, or iron deficiency, are required to assess the effect of GSM powder on iron status markers. It should be noted that the level of CRP was elevated in both GSM and placebo groups during the study and this effect was possibly confounded by the COVID-19 vaccination of study participants.

The results of this study should be interpreted with caution because of several limitations. Firstly, although randomisation was stratified based on BMI, a somewhat higher mean BF% was found in the GSM group compared with placebo, but as this was taken into consideration in data analysis, it is unlikely to have impacted the results. Secondly, it was impossible to consider the inclusion of a controlled diet to reduce inter-subject variation in dietary intake and microbiota over time. Moreover, it is important to note that some of the participants took medications prescribed to them by their physician for treating chronic conditions that had been administered to them for long periods of time before participating in the trial and were maintained as such during the trial. Therefore, it is likely that the baseline abundance of microbiota was not influenced by these medications over the 12 weeks.

In conclusion, GSM supplementation had minor effects on the abundance of some of the commensal bacteria such as *Bacteroides* and *Bifidobacterium* which tended to increase over the 12-week study period. Iron status markers and body composition were not notably affected by GSM treatment; however, the results of this study do contribute to the knowledge surrounding the effects of whole GSM powder on these outcome measures in healthy postmenopausal women.
